# Is educational attainment related to end-of-life decision-making? A large post-mortem survey in Belgium

**DOI:** 10.1186/1471-2458-13-1055

**Published:** 2013-11-09

**Authors:** Kenneth Chambaere, Judith AC Rietjens, Joachim Cohen, Koen Pardon, Reginald Deschepper, H Roeline W Pasman, Luc Deliens

**Affiliations:** 1 End-of-life Care Research Group, Vrije Universiteit Brussel & Ghent University, Laarbeeklaan 103, Brussel 1090, Belgium; 2 Department of Public and Occupational Health, EMGO Institute for Health and Care Research, VU University Medical Centre, Amsterdam, The Netherlands

**Keywords:** Educational attainment, Socio-economic inequalities, Cultural capital, End-of-life care, End-of-life decisions, Euthanasia

## Abstract

**Background:**

Educational attainment has been shown to influence access to and quality of health care. However, the influence of educational attainment on decision-making at the end of life with possible or certain life-shortening effect (ELDs ie intensified pain and symptom alleviation, non-treatment decisions, euthanasia/physician-assisted suicide, and life-ending acts without explicit request) is scarcely studied. This paper examines differences between educational groups pertaining to prevalence of ELDs, the decision-making process and end-of-life treatment characteristics.

**Method:**

We performed a retrospective survey among physicians certifying a large representative sample of Belgian deaths in 2007. Differences between educational groups were adjusted for relevant confounders (age, sex, cause of death and marital status).

**Results:**

Intensified pain and symptom alleviation and non-treatment decisions are more likely to occur in higher educated than in lower educated patients. These decisions were less likely to be discussed with either patient or family, or with colleague physicians, in lower educated patients. A positive association between education and prevalence of euthanasia/assisted suicide (acts as well as requests) disappeared when adjusting for cause of death. No differences between educational groups were found in the treatment goal in the last week, but higher educated patients were more likely to receive opioids in the last day of life.

**Conclusion:**

There are some important differences and possible inequities between educational groups in end-of-life decision-making in Belgium. Future research should investigate whether the found differences reflect differences in knowledge of and adherence to patient preferences, and indicate a discrepancy in quality of the end of life.

## Background

Socio-economic status has been consistently identified as a significant contributor to differences in use, access and quality of health care [1,2]. Social background, occupation, income and education –indicators of overall socio-economic status – are thought to influence both the patient’s inclination to seek proper preventive and treatment care and how the health care system treats its patients [3,4]. This is contradictory to the widely held principle of equal access to and distribution of public health care regardless of social, cultural or economic factors [5].

One area of medical care which has been scarcely studied for socio-economic differences and possible inequalities is end-of-life care, particularly decision-making at the end of life. In this paper we survey the relationship between educational attainment and the occurrence and decision-making process of end-of-life decisions *with a possible or certain life-shortening effect* (ELDs) in Flanders, Belgium. ELDs are subdivided into intensified alleviation of pain and symptoms, non-treatment decisions, euthanasia/physician-assisted suicide and life-ending acts without explicit request.

Euthanasia, ie administration of lethal drugs with the explicit intention of hastening death at the explicit request of the incurable patient, was decriminalised in Belgium in 2002 under strict legal terms [6]. Sceptics anticipate this will lead to the violation of the rights particularly of vulnerable patient groups, ic the least educated, resulting in disproportionately more non-voluntary “mercy killing” in these patients (termed the slippery slope argument) [7-9]. Following the euthanasia law, laws on patient rights and on palliative care were also passed in 2002. In short, the Law on Patient Rights prescribes quality health service to all patients, including the right to information about treatment and informed consent [10]. The Law on Palliative Care ordained that every patient has the right to adequate palliative care at the end of life [11]. Within these laws is embedded the principle of equality in health care, applicable to the end of life. But do these laws actually achieve equality – ic between educational groups – in end-of-life care?

The specific research questions are the following: 1) are there differences in the prevalence of ELDs between educational groups?; 2) are there differences in the decision-making process (patient, family and caregivers) and end-of-life treatment between educational groups?

In explaining the impact of educational attainment on health-related outcomes, mediating factors such as material and (psycho)social resources are cited [12,13], but also specific cultural resources (values, attitudes, beliefs, knowledge, skills, communication competencies, empowerment, self-advocacy and self-determination) have been posited as being important [14-16]. All these factors may influence the frequency and manner in which ELDs are made and lead to inequalities between educational groups.

Scientific literature has either identified or predicted a number of inequalities in end-of-life care and decision-making according to educational level. Following hypotheses are relevant to this study: a) euthanasia will be more prevalent among the higher educated [17-19] given their higher degree of acceptance [20] and tendency towards more self-determination and autonomy [14-16]; b) life-ending acts without explicit request will be more frequent among the least educated (ie the slippery slope hypothesis) [7-9]; c) due to less desired aggressiveness of treatment at the end of life, highly educated patients will more often forgo life prolonging treatment [21-23]; d) supposing better competencies, communication skills, knowledge of their rights and more appeal to autonomy, highly educated patients and their families will be more often involved in decision-making [24-28]; and e) through this higher degree of involvement and self-advocacy, highly educated patients may achieve better palliative treatment of pain and symptoms [28,29].

## Methods

### Death certificate survey

We performed a post-mortem survey of physicians certifying a large and representative sample of all deaths in 2007 in Flanders, a semi-autonomous region in Belgium with approximately six million inhabitants and 55,000 deaths annually. The stratified random sample was drawn at the Flemish Agency for Care and Health, central administration authority for processing death certificates. All deaths from June until November 2007 of Belgian residents aged one year or older were assigned to one of four strata, based on underlying cause of death as indicated on the death certificate and the estimated corresponding likelihood of an ELD. Sampling fractions for each stratum increased with this likelihood. This resulted in a sample of 6927 deaths, about 25% of all deaths in the studied months and 12% of all deaths in 2007.

Certifying physicians were sent a five-page questionnaire for maximum five patients, with at most three reminders in case of non-response. A lawyer was involved in the mailing procedure as intermediary between responding physicians, researchers and the Flemish Agency for Care and Health to guarantee that completed questionnaires could never be linked to a particular patient or physician. By guaranteeing anonymity the potential for social desirability bias was decreased. After data collection a one-page questionnaire was mailed to all non-responding physicians, asking for the reasons for not participating. The mailing and anonymity procedure were approved by the ethical review committees of the university hospitals of the Vrije Universiteit Brussel and Ghent University, by the Belgian National Disciplinary Board of Physicians, and by the Federal Privacy Commission. The study design, sampling, and mailing procedure are described in detail elsewhere [30].

### Questionnaire

The questionnaire was validated through testing by a panel of physicians. It first asked whether death had been sudden and unexpected. If this question was answered negatively (and hence an ELD prior to death would not be precluded) physicians were asked whether they had: withheld or withdrawn medical treatment taking into account or explicitly intending hastening of the patient’s death; intensified the alleviation of pain and/or other symptoms with drugs taking into account or co-intending the possible hastening of death; and administered, supplied, or prescribed drugs with the explicit intention of hastening death. If in the latter case the drugs had been administered by someone other than the patient at the patient’s explicit request or prescribed/supplied and self-administered, it was classified as *euthanasia/assisted suicide*. If there had been no explicit request from the patient, the act was classified as a *life ending act without explicit patient request*. Regardless of whether an ELD was made, an additional question was posed for every non-sudden death whether the patient had made a request for euthanasia that had not been granted.

More than one ELD can be made in the same patient. Because having the physician answer the same questions about the process of every decision would excessively burden the respondent, we elected to pose such questions solely for the most important decision. We defined this as the decision with the most explicit life-shortening intention, and where two decisions with similar life-shortening intention were made, administering drugs prevailed over withholding or withdrawing treatment. Questions on decision-making included discussion with the patient, family and other caregivers, whether the patient had made a request, whether the patient had been deemed competent by the physician and whether the patient had ever, implicitly or explicitly, expressed a wish for life-ending. Demographic and clinical patient data were obtained from the death certificates, and linked anonymously after data collection. All information found in the death certificates used in this study is provided by the certifying physician, with the exception of highest education which is provided by officials of the municipality who have access to a national registry of socio-economic data.

### Analysis

**Figure 1:**
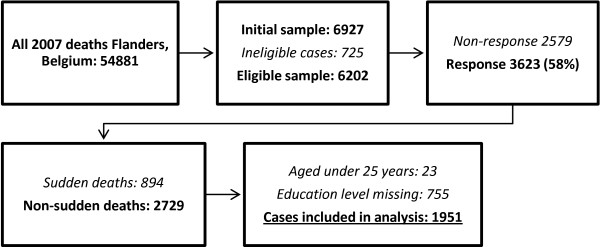
**Flow chart of the death certificate survey in Flanders**, **Belgium.** The initial sample was 6927 of all 54881 deaths in 2007. From non-response analyses we found that response was impossible for 725 deaths eg because the physician did not have access to the patient’s medical file or the patient could not be identified. In total 3623 were returned and the response rate was 58.4% (3623/6202 eligible cases). We selected non-sudden deaths as denominator in all analyses. Considering the educational trajectory is likely not complete in minors and early adults, we elected a cut-off age of 25 years (23 cases deleted). Of the remaining non-sudden deaths, 755 cases (27.7%) had missing values for educational attainment and were discarded. This resulted in a final sample of 1951 patients.

From non-response analyses we found that response was impossible for 725 deaths eg because the physician did not have access to the patient’s medical file or the patient could not be identified. In total 3623 were returned and the response rate was 58.4% (3623/6202 eligible cases). The response sample was in a first step corrected for disproportionate stratification (by assigning weights inverse to the strata sampling fractions) and in a second step adjusted to be representative of all 2007 deaths for age, sex, place and cause of death (case weights calculated to reflect the distribution and combination of these characteristics in all 2007 deaths). After this complex weighting procedure there were no significant differences between our response sample and the entire “population” of 2007 deaths (for the combination of age, sex, cause of death and place of death). All analyses are done with weighted data. We selected non-sudden deaths as denominator in all analyses. Considering the educational trajectory is likely not complete in minors and early adults, we elected a cut-off age of 25 years (23 cases deleted). Of the remaining non-sudden deaths, 755 cases (27.7%) had missing values for educational attainment and were discarded. This resulted in a final sample of 1951 patients. Figure 1 presents the survey’s flow chart. Multivariate analysis for differences in sociodemographic and clinical characteristics and dependent variables between the study cases and the cases for which educational attainment was missing, showed significant differences only in cause of death (more deaths from cardiovascular disease in study cases, p .007) (not in figure).

The initial 11-category variable for educational attainment was recoded into three categories yielding high case count categories: none/primary, lower secondary and higher secondary/higher. Bivariate percentages were calculated and adjusted for age and sex, through direct standardisation with all non-sudden deaths as standard population, and tested with χ^2^ test. Logistic regressions (with educational attainment, age, sex, marital status and cause of death) were performed to determine multivariate p-values, odds ratios and 95% confidence intervals. A p-value < .05 is considered to indicate statistical significance. All statistical analyses were done using SPSS 19.0.

## Results

The 2007 sample characteristics by educational attainment are shown in Table 1. Educational attainment is associated with all presented characteristics (p < .001). Patients with no or primary education are more often old, female, widowed, dying from cardiovascular or respiratory disease and in a care home than the higher educated. These differences between educational groups indicate the need for a controlling for these confounders when comparing both groups for end-of-life decision-making.

**Table 1:** Sample characteristics of non-sudden deaths by educational attainment (weighted %)

	**Total**	**Educational attainment**			
		**None/**	**Lower**	**Higher**	**χ**^**2**^
		**primary**	**secondary**	**secondary/**	**p-value**
				**higher**	
Unweighted n	1951	877	530	544	
Weighted %	100	49.7	25.6	24.7	
**Age (years)**					*<.001*
25-64	15.9	6.2	17.5	33.6	
65-79	33.7	28.6	41.7	35.8	
80+	50.4	65.2	40.8	30.6	
**Sex**					*<.001*
Male	48.8	38.8	55.9	61.6	
Female	51.2	61.2	44.1	38.4	
**Marital status**					*<.001*
Married	47.6	36.2	55.3	62.7	
Unmarried/divorced	14.6	12.7	14.5	18.6	
Widowed	37.8	51.1	30.3	18.6	
**Cause of death**					*<.001*
Cancer	35.3	26.5	42.5	45.5	
Cardiovascular	30.8	35.3	27.6	25.2	
Respiratory	12.1	14.6	10.7	8.4	
Neurological	4.1	4.0	3.1	5.5	
Other	17.7	19.7	16.0	15.5	
**Place of death**					*<.001*
Hospital	53.0	49.5	54.3	58.5	
At home	19.9	16.6	22.0	24.1	
Care home	24.6	31.9	19.8	15.0	
Other	2.5	1.9	4.0	2.3	

Table 2 shows the prevalence of ELDs by educational attainment, adjusted for age and sex. Intensified alleviation of pain and symptoms with life-shortening co-intended is found to occur more often among higher educated groups (12.7% and 13.2% vs. 7.6%), both in cancer and non-cancer patients (data not shown). This result holds when controlling for other confounders (marital status and cause of death): both lower secondary and higher (secondary) education had higher odds ratios compared to no or primary education (1.72 and 1.51 respectively). The significant age and sex standardized differences for non-treatment decisions also hold when controlling for other confounders, but were only significant for the highest educated group (1.44) as compared to the lowest. The higher educated groups were significantly more likely to receive euthanasia (3.9% and 3.8% vs. 1.6%), and they also formulated a request more often (5.4% and 6.2% vs. 3.3%). This difference between educational groups was found only in cancer patients, not in non-cancer patients (data not shown). Controlling for other confounders, only patients with lower secondary education have a higher chance (2.31) of euthanasia than patients with no or primary education, and prevalence of request or granted requests did not differ significantly between educational groups. There was no association between educational attainment and other ELDs.

**Table 2:** ELD prevalence by educational attainment, non-sudden deaths*

	**Educational attainment**						
			**Higher**		**Multivariate**		
	**None/**	**Lower**	**secondary/**		**OR (95% CI)**	**OR (95% CI)**	**p-value**
	**primary**	**secondary**	**Higher**	**χ**^**2**^	**Lower secondary**	**Higher (secondary)**	
	**n = 877**	**n = 530**	**n = 544**	**p-value**	**vs. none/primary**	**vs. none/primary**	
APS-	39.8	43.2	40.6	.486	1.01 (0.79-1.29)	1.00 (0.77-1.29)	.996
APS+	7.6	13.2	12.7	*.001*	*1.74 (1.20-2.53)*	*1.52 (1.01-2.28)*	*.012*
NTD-	48.7	49.6	57.4	*.009*	1.08 (0.85-1.36)	*1.45 (1.13-1.86)*	*.013*
NTD+	15.3	17.5	18.1	.336	1.25 (0.91-1.71)	1.32 (0.94-1.83)	.204
EAS	1.6	3.8	3.9	*.016*	*2.36 (1.07-5.19)*	1.88 (0.83-4.26)	.103
…requested	3.3	6.2	5.4	*.034*	1.58 (0.89-2.78)	1.40 (0.77-2.53)	.280
…(granted)	(49)	(62)	(72)	.246	2.86 (0.78-10.8)	1.70 (0.47-6.21)	.284
LAWER	2.8	3.0	2.2	.749	0.96 (0.48-1.95)	0.90 (0.41-1.98)	.966

*Figures are percentages adjusted for age and sex, with all non-sudden deaths as standard population, and weighted for representativeness.

More than one end-of-life decision possible for one case.

The p-values in italic indicate significant differences between educational level groups (p < .05, no Bonferoni correction). Variables entered in multivariate logistic regression model: educational attainment, age, sex, marital status, cause of death; no interaction effects.

ELD: end-of-life decision; APS-: intensified alleviation of pain and symptoms taking possible life-shortening into account; APS+: APS with life-shortening co-intended; NTD-: non-treatment decision taking possible life-shortening into account; NTD+: NTD with life-shortening explicitly intended; EAS: euthanasia/assisted suicide; LAWER: life-ending acts without explicit patient request.

Physicians discussed non-treatment decisions with the patient less often when that patient had no or primary education (adjusted for age and sex differences), but the effect disappeared when controlling for other confounders (Table 3). After controlling for these confounders the likelihood was higher in the least educated group as compared to the higher educated groups that neither the patient nor the family had been consulted for intensified pain and symptom alleviation and non-treatment decisions, and that no colleague physician had been consulted. Also for euthanasia discussion with a colleague was found more often in highly educated groups, although this did not hold after controlling for other confounders.

**Table 3:** Decision making characteristics by most important ELD and educational attainment*

	**Intensified alleviation of pain and symptoms**			**Non**-**treatment decisions**			**Euthanasia**/**assisted suicide**			**Life**-**ending without explicit request**		
			**Higher**	**none/**	**Lower**	**Higher**			**Higher**	**None/**	**Lower**	**Higher**
	**None/**	**Lower**	**secondary/**	**primary**	**secondary**	**secondary/**	**None/**	**Lower**	**secondary/**	**primary**	**secondary**	**secondary/**
	**primary**	**secondary**	**Higher**			**Higher**	**primary**	**secondary**	**higher**			**higher**
	**n = 380**	**n = 257**	**n = 263**	**n = 196**	**n = 95**	**n = 108**	**n = 17**	**n = 42**	**n = 39**	**n = 27**	**n = 9**	**n = 12**
Discussed with patient**	21	28	24	*15*	*28*	*16*	100	100	100	18	45	31
…and explicit request by patient	15	17	18	10	6	10	100	100	100	0	0	0
Not discussed with patient	79	72	76	*85*	*72*	*84*	0	0	0	82	55	69
…but patient competent	10	16	11	3	2	7	-	-	-	4	0	7
…but ever wish stated by patient	9	14	13	*16*	*10*	*24*	-	-	-	*44*	*14*	*0*
…but discussed with family	44	44	48	52	49	57	-	-	-	76	29	63
Discussed with patient nor family	35	28	28	33	22	26	0	0	0	6	27	7
Discussed with colleague(s)	33	38	42	45	57	55	*42*	*70*	*86*	64	47	42
Discussed with PC specialist	21	22	28	12	12	21	30	52	51	7	18	11

*Figures are percentages adjusted for age and sex, expressed to all non-sudden deaths, and weighted for representativeness.

End-of-life decisions in this table are the most important decision, ie only one decision per death.

Percentages in italic denote significant (χ^2^) differences between education levels, underlined percentages indicate multivariately significant differences (p < .05, no Bonferoni correction). Variables entered in multivariate logistic regression model: educational attainment, age, sex, marital status, cause of death; no interaction effects.

PC: palliative care.

**euthanasia and physician-assisted suicide are by definition always discussed with the patient.

No differences between education levels were found in the (palliative) care orientation in the last week of life (Table 4). However, there was a significant relationship with opioids administered in the last 24 hours independent of other sociodemographic or clinical characteristics: compared to patients with no or primary education, higher educated groups had 1.44 and 1.47 higher odds to receive opioids in the last 24 hours. This significant relationship was found in both cancer and non-cancer patients (data not shown).

**Table 4:** **End**-**of**-**life treatment characteristics by educational attainment**, **non**-sudden deaths*

	**Educational attainment**						
	**None/**	**Lower**	**Higher**		**Multivariate**		
	**primary**	**secondary**	**secondary/**		**OR (95% CI)**	**OR (95% CI)**	**p-value**
			**higher**	**χ**^**2**^	**lower secondary**	**higher (secondary)**	
	**n = 877**	**n = 530**	**n = 544**	**p-value**	**vs. none/primary**	**vs.none/primary**	
Palliative care goal in last week**	72.9	77.6	72.1	.168	1.18 (0.86-1.61)	0.84 (0.61-1.16)	.167
Opioids administered in last 24 hours	57.3	66.7	66.0	*<.001*	*1.42 (1.10-1.84)*	*1.49 (1.13-1.96)*	*.005*

*Figures are percentages adjusted for age and sex, expressed to all non-sudden deaths, and weighted for representativeness.

**as opposed to life prolongation or curation.

The p-values in italic indicate significant differences between educational level groups (p < .05, no Bonferoni correction). Variables entered in multivariate logistic regression model: educational attainment, age, sex, marital status, cause of death; no interaction effects.

## Discussion

This study found a number of differences in end-of-life decision-making between educational groups, both in the occurrence and in the decision-making process. The hypotheses formulated at the outset of this paper can be carefully scrutinised using presented data.

The first hypothesis expected that euthanasia is more prevalent among highly educated patients given their more positive attitude towards euthanasia and tendency to self-determination and autonomy [14-20]. We indeed found a higher prevalence of euthanasia performance and requests in higher educated patients, adjusted for age and sex. Though the association was strong, it largely disappeared in multivariate analysis with cause of death (cancer) as principal confounder. As Table 1 showed, the higher educated groups died more often with cancer than the lower educated. Cancer has in previous studies been found to be strongly associated with euthanasia because it is characterized by a high and often unbearable symptom burden and a relatively predictable end-of-life trajectory compared to for instance cardiovascular or respiratory disease, allowing for better anticipation of deteriorating functional status [31]. In any case, in this and previous studies educational level was not found to affect euthanasia prevalence unequivocally [18,19,32,33]; the hypothesis and underlying rationale are not conclusively confirmed here. However, the lack of an unambiguous independent effect may be due to the low number of patients. The idea of education and particularly accompanying attitudes and competencies influencing euthanasia prevalence should not be abandoned.

The second hypothesis that problematic life-ending acts without explicit request will be found more often in the least educated [7-9] was not confirmed by our results. The prevalence and odds ratios did not differ significantly between educational groups. Also other ELDs that could be regarded unethical, ie where the physician reported a life-shortening intent in intensified alleviation of pain and symptoms or non-treatment decisions, were not found more often in the least educated, rather to the contrary. The data thus seem to contradict the ‘slippery slope’ hypothesis that physician assisted dying, once regulated, will disproportionately affect vulnerable patient groups negatively [7-9], and corroborates other studies in countries with euthanasia regulation [33-36]. If anything, trend studies before and after legalisation have shown that end-of-life practice has generally improved since legalisation, without reported inequalities for any of the supposed vulnerable groups [33-35]. However, as adverse effects may only become noticeable after some time or the effect may be more subtle, it is necessary to continue monitoring these highly controversial decisions.

In our third hypothesis we expected non-treatment decisions to occur more often in the highest educated due to their lower inclination towards aggressive end-of-life treatment [21-23]. The tendency was indeed towards more withheld/withdrawn treatment in the highest educated group compared to the lowest educated group. One could expect that the higher educated generally demand more comprehensive treatment when ill, but at the end of life when cure is no longer expected, this is not the case. Although we have no information on the motivations of the patients this difference could be related to the higher educated groups being more concerned with self-determination and control over the end of life. There can also be an influence of a higher degree of assertiveness to go against physicians’ recommendations to exhaust life-prolonging treatment options. Indeed, prior research found more use of palliative care services [28] and more advance care planning [24,37,38] in the higher educated. Future studies should investigate these explanations further, and perhaps differentiate between the types of forgone treatments (eg mechanical ventilation, CPR, antibiotics, hydration and tube feeding, etc.).

The fourth hypothesis predicted a higher degree of patient involvement in end-of-life decision-making for the higher educated [24-28]. This relationship could be seen in the age and sex adjusted analyses of intensified alleviation of pain and symptoms and non-treatment decisions but disappeared when controlling for other confounders. However, looking at discussion with either patient or family, we did see an independent effect of education, hinting toward more communication, better competencies, less “distance of power” and less paternalism in the higher educated [24-28,38]. Higher educated patients are thought to be better able to speak in a proto-professionalised manner, which appeals more to their physicians [39]. Lower educated patients may prefer not to be included in making such difficult decisions and rather trust to their physician’s judgment.

Another striking finding is that physicians consulted with colleagues about ELDs more often when it concerned higher educated patients. Perhaps discussion with patient and family prompts physicians to consult with colleagues, as especially highly educated patients may demand a thoroughly substantiated discourse, with more than one physician and professional opinion. Or possibly the mere awareness or perception of a patient being higher educated and more empowered may cause physicians to conduct the decision-making process more thoroughly.

Our fifth and final hypothesis, predicting better palliative treatment of pain and symptoms for the higher educated [28,29], also seems to be reflected in the data: though no difference was found in the palliative treatment goal in the last week, highly educated patients more often received opioids in the final 24 hours than did lowly educated patients, and their pain and symptom treatment is more often intensified to the point that life-shortening is accepted. As these results are controlled for sociodemographic and clinical characteristics, the explanation is likely to lie within the physician-patient relationship, more specifically in the previously mentioned competence in higher educated patients to be self-advocating and to better voice their needs and preferences. This finding is again cause for concern as it implies a difference in quality of end-of-life care between educational groups.

Some study limitations have to be taken into account. First, the education variable had many missing cases (27.6%), which strongly reduced the statistical power of the analyses, but probably did not impact the representativeness of the results. Second, we have no information about patients’ preferences and the contents of end-of-life discussions, which may differ across educational groups [40] and could have provided invaluable insight into the found differences.

## Conclusions

From our data an overall picture emerges that patients’ education has a significant influence on their end-of-life care and decision-making processes. We have explained this through mediating factors often referred to as “cultural resources” or “cultural capital” [14-16]: patients with a higher educational attainment are thought to be more empowered, assertive and self-advocating, putting more emphasis on self-determination and control over the end of life, and being more insistent on thorough end-of-life care and decision-making. They are also projected to have better knowledge, skills and communication competencies to enforce these wishes. Health policy should address this issue and sensitise health care workers about legal patient rights requirements. Physicians should be encouraged to involve patients who are less empowered. In situations where patients do not initiate discussions themselves, physicians should be the ones to take the first step. These findings complement other research uncovering health-related differences based on educational attainment through other mechanisms such as material, (psycho)social and behavioural differences [12,13].

We conclude that there are some important differences and possible inequities between educational groups in end-of-life decision-making in Belgium. Future research should focus on a better understanding of the detailed causes and reasons of these differences.

## Abbreviations

APS-: Intensified alleviation of pain and symptoms taking possible life-shortening into account; APS+: APS with life-shortening co-intended; EAS: Euthanasia/assisted suicide; ELDs: End-of-life decisions with a possible or certain life-shortening effect; LAWER: Life-ending acts without explicit patient request; NTD-: Non-treatment decision taking possible life-shortening into account; NTD+: NTD with life-shortening explicitly intended; PC: Palliative care.

## Competing interests

The authors declare that they have no competing interests.

## Authors’ contributions

All authors (1) made significant contributions to conception and design, or acquisition of data, or analysis and interpretation of data; (2) were involved in drafting the manuscript or revising it critically for important intellectual content; and (3) have given final approval of the version to be published.

## Pre-publication history

The pre-publication history for this paper can be accessed here:

http://www.biomedcentral.com/1471-2458/13/1055/prepub
